# Influencing factors of recurrence after pelvic organ prolapse surgery and construction of a nomogram risk prediction model

**DOI:** 10.1590/1806-9282.20240849

**Published:** 2024-12-02

**Authors:** Penghui Zhang, Weijie Du, Gang Guo, Meijuan Yuan, Jun Wei

**Affiliations:** 1 Zhejiang Chinese Medical University – Hangzhou, China.; 2 Shulan (Hangzhou) Hospital Affiliated to Zhejiang Shuren University, Shulan International Medical College, Department of Gynecology – Hangzhou, China.

**Keywords:** Pelvic organ prolapse, Postoperative, Recurrence, Prediction, Risk factors

## Abstract

**OBJECTIVE:**

Pelvic organ prolapse affects women’s quality of life through symptoms such as vaginal laxity, urinary incontinence, defecation and sexual dysfunction, and pelvic pain. Given the challenges in managing recurrent cases, understanding risk factors and the effect of surgical choices on recurrence is vital for guiding clinical decisions. This study explores how uterine preservation influences postoperative recurrence and develops predictive models to aid in assessing recurrence risk.

**METHODS:**

A total of 87 patients diagnosed with pelvic organ prolapse who underwent laparoscopic sacral fixation were included. Patients were classified into two groups based on the occurrence of pelvic organ prolapse recurrence within 3 years post-surgery (recurrence: n=22; no recurrence: n=65). Follow-up over 3 years was recorded. Factors including age, body mass index, birth order, occupation, and uterus preservation during surgery were evaluated. The relationship between pelvic floor muscle strength and pelvic organ prolapse recurrence was also examined. Logistic regression analysis assessed the correlation between pelvic organ prolapse recurrence and levels of serum elastase inhibitor and osteopontin.

**RESULTS:**

In a follow-up of 87 patients with pelvic organ prolapse, 22 experienced recurrences within 3 years, marking a 25.29% recurrence rate. Multivariate analysis identified older age, higher parity, and sustained contraction of type II muscle fibers as independent risk factors for recurrence (all p<0.05). Lower systolic blood pressure in type I and II muscle fibers was associated with decreased serum elastase inhibitor and osteopontin levels, increasing pelvic organ prolapse recurrence risk. Logistic regression identified age, multiple deliveries, and low systolic pressure in type II muscle fibers as independent recurrence factors. The constructed nomogram risk prediction model, incorporating these factors, showed good discrimination ability with an area under the receiver operating characteristic curve of 0.891 (95%CI 0.871, 0.921), indicating accurate predictions and high net benefit.

**CONCLUSION:**

Factors such as age, birth order, uterine preservation, and pelvic floor muscle strength impact postoperative pelvic organ prolapse recurrence. Older age, a higher number of deliveries, and reduced systolic pressure of class II muscle fibers are independent risk factors for pelvic organ prolapse recurrence after surgery.

## INTRODUCTION

Pelvic organ prolapse (POP) is a condition resulting from various factors that cause significant damage to the pelvic floor muscles. It commonly presents clinical symptoms such as vaginal laxity, pelvic organ prolapse, stress urinary incontinence, defecation dysfunction, sexual dysfunction, and pelvic pain, all of which severely impact women’s quality of life^1^. Currently, sacropexy is the preferred treatment option for POP, with surgical approaches classified into two categories: preserving the uterus or performing a hysterectomy. However, there is an ongoing debate regarding the preservation of the uterus and its potential correlation with an increased risk of postoperative recurrence in POP patients^2,3^. Recent research revealed the challenges in re-treating women with POP after recurrence, indicating that secondary surgery may encounter specific complications, such as problems with scar tissue, tissue adhesions, and unclear anatomical layers, which increase the difficulty of surgery and increase the risk of surgical failure^4^. Consequently, it is crucial to identify the risk of recurrence in POP patients before surgery and comprehend the impact of surgery on recurrence in order to guide clinicians in formulating surgical plans and implementing necessary measures. A reliable risk prediction model is essential to aid in preoperative planning and patient counseling, helping to optimize surgical outcomes and reduce the incidence of postoperative recurrence. This not only holds significance for the prevention and treatment of postoperative recurrence in POP patients but also provides valuable insights for clinical decision-making. Currently, most studies only describe the recurrence of POP patients, with little exploration of its contributing factors. Hence, this study aimed to investigate the influence of uterus preservation on postoperative recurrence in POP patients, examine the factors influencing recurrence, and establish predictive models to facilitate the clinical evaluation of postoperative recurrence risk.

## PATIENTS AND METHODS

### Subjects

A total of 87 POP patients undergoing surgery from January 2020 to January 2023 were enrolled. Inclusion criteria were as follows^3^: (1) meeting POP diagnostic criteria; (2) pelvic floor muscle strength Grade III or below; (3) laparoscopic sacral fixation surgery; and (4) good compliance for follow-up. Exclusion criteria were as follows^3^: (1) neurological disease or dysfunction; (2) severe uterine malformation; (3) prior reproductive surgery; and (4) non-compliance with treatment. All participants provided written informed consent. The study was approved by the hospital’s medical ethics committee.

### Methods

#### Surgical methods

##### Uterosacral fixation with uterus preservation

Under general anesthesia, a uterine elevator was inserted and an endoscopy was performed. The retroperitoneum was opened from the sacral promontory to near the right ureter, exposing the vascular area before the first sacral vertebra and the right sacral ligament to the posterior vaginal fornix. The rectovaginal space was separated and the bladder pushed downward, exposing about 3–4 cm of the anterior vaginal wall. A hole was made in the right broad ligament above the blood vessels, and a Y-shaped mesh was placed around the cervix. The mesh arms were sutured to the anterior cervix and vaginal wall, the cervical ring, and the posterior vaginal wall. The other end was fixed to the anterior longitudinal ligament in front of the first sacrum. Finally, absorbable thread was used to suture the mesh behind the peritoneum, and the abdomen was sequentially closed.

##### Hysterectomy vaginosacralization

Following successful administration of general anesthesia, the patient underwent a procedure involving the placement of a uterine elevator and endoscope. The retroperitoneum was then accessed by making an incision near the right ureter and the sacral promontory, allowing for exposure of the vascular region before the first sacral vertebra. Subsequently, the lateral peritoneum was opened along the right sacral ligament, reaching the posterior vaginal fornix. A total hysterectomy was performed, followed by suturing of the vaginal stump. The vaginal bladder space and vaginal rectal space were carefully separated. A Y-shaped mesh was utilized, and both arms were sutured and secured to the fibromuscular layer of the anterior and posterior walls of the vagina using non-absorbable thread. At the other end, the mesh was sutured and anchored to the anterior longitudinal ligament of the first sacral anterior bloodless area, ensuring there was no tension. The retroperitoneum was sutured with absorbable thread, with the mesh being completely positioned behind the peritoneum. Finally, the abdomen was closed in a sequential manner.

##### Grouping

Based on the occurrence of recurrence within a 3-year period following the surgery, the patients were classified into two groups: those who experienced recurrence (n=22) and those who did not (n=65). The definition of POP recurrence was determined based on the criteria established by the Network of Pelvic Floor Diseases. In other words, the presence of POP recurrence could be confirmed by meeting any of the following conditions: (1) if the POP-Q stage indicator point exceeds the hymen margin or (2) if the patient’s quality of life is affected by vaginal bulge symptoms^5^.

#### Observed indicators

##### General clinical data

The study gathered information regarding the patients’ age, body mass index (BMI), birth order, and occupation. Additionally, the surgical approach was documented, noting whether the preservation of the uterus was performed or not.

##### Pelvic floor muscle strength

The assessment of pelvic floor muscle strength was conducted using the PHENIX USB4 neuromuscular stimulator from Bohua Medical Equipment Co., Ltd. To evaluate the muscle strength of type I and type II muscle fibers, measurements of continuous systolic pressure and contraction duration were taken.

##### Serum biochemical indicators

In the morning, a fasting venous blood sample of 3 mL was extracted from the patients. Following the centrifugation process, the resulting supernatant was isolated for further analysis. ELISA was utilized to measure the concentrations of serum elastase inhibitor (also known as elastase inhibitor or Elafin) and osteopontin (OPN). Prior to the surgical procedure, a comparison was made between the levels of Elafin and OPN in the recurrent group and the non-recurrent group.

##### Influencing factors of recurrence after pelvic organ prolapse

Multivariate analysis was conducted to examine the demographic characteristics, pelvic floor muscle strength index, and serum biochemical index in order to identify the distinguishing factors between the recurrent and non-recurrent groups. Additionally, an investigation was carried out to determine the independent factors that contribute to the recurrence of POP.

#### Statistical analysis

Statistical analysis was performed using the SPSS 25.0 and R 4.3.1 software. The χ^2^ test and two independent sample t-tests were used to compare the groups. Logistic regression analyzed factors influencing POP recurrence. Line chart prediction models, receiver operating characteristic (ROC), calibration, and decision curve analysis (DCA) curves were generated. Differences were considered statistically significant (p<0.05).

## RESULTS

### Recurrence after pelvic organ prolapse

Of the 87 POP patients followed up, 22 patients had recurrence within 3 years, with a recurrence rate of 25.29% (22pm 87).

### Comparison of recurrence in pelvic organ prolapse patients with different characteristics

The study aimed to compare the rate of recurrence among patients with POP based on factors such as age, occupation, BMI, parity, and operation methods (Table 1). The findings revealed that older age, higher parity, and the preservation of the uterus were associated with a higher recurrence rate in POP patients. Moreover, patients with lower levels of continuous systolic pressure in type I and II muscle fibers, as well as lower levels of serum Elafin and OPN before the operation, exhibited a higher likelihood of postoperative recurrence. However, no significant correlation was observed between BMI, occupation, duration of pelvic floor muscle fiber contraction, and postoperative recurrence in patients with POP.

**Table 1:** Baseline characteristics.

Index	Non-recurrent group (n=65)	Relapse group (n=22)	x^2^/t	p
Age (`χ±s, years)	52.78±6.04	62.77±5.96	6.728	<0.001
BMI (`χ±s, kg/m^2^)	21.58±2.78	21.10±1.27	1.095	0.277
Occupation [n (%)]				
Farmers	48 (73.85)	21 (95.45)	5.139	0.077
Public officer	11 (16.92)	0		
Other	6 (9.23)	1 (4.55)		
Number of births (`χ±s, times)	2.38±0.84	4.00±1.57	4.597	<0.001
Mode of operation [n (%)]			6.396	0.011
Preserve the uterus	27 (41.54)	16 (72.73)		
Excision of uterus	38 (58.46)	6 (27.27)		
Continuous systolic blood pressure of type I muscle fibers (`χ±s, kPa)	2.49±0.17	2.14±0.15	8.901	<0.001
Contraction duration of type I muscle fibers (`χ±s, s)	3.68±1.11	3.32±0.95	1.362	0.177
Continuous systolic blood pressure of type II muscle fibers (`χ±s, kPa)	4.43±0.15	4.10±0.15	9.278	<0.001
Contraction duration of type II muscle fibers (`χ±s, s)	2.80±0.62	3.14±0.99	1.498	0.146
Serum Elafin level (`χ±s, pg/mL)	7.31±0.13	7.21±0.18	2.266	0.031
Serum OPN level (`χ±s, mg/L)	9.27±0.12	9.02±0.11	8.467	<0.001

BMI: body mass index; Elafin: elastase inhibitor; OPN: osteopontin.

### Multivariate analysis of recurrence after pelvic organ prolapse

The dependent variable in this study was the recurrence of POP among postoperative patients, categorized as either recurrence (1) or non-recurrence (0). The independent variables included age, parity, mode of operation (preservation of uterus or not), continuous systolic blood pressure of type I and II muscle fibers, serum Elafin level, and serum OPN level. These variables were selected based on their statistical significance in the previous univariate analysis. To determine the independent factors associated with recurrence, a multivariate logistic regression model was employed. The findings revealed that older age, higher parity, and sustained contraction of type II muscle fibers were identified as independent risk factors for the recurrence of POP (all p<0.05). However, the preservation of the uterus during the operation was not found to be an independent risk factor for the recurrence of POP, as indicated in Table 2.

**Table 2:** Logistic analysis of influencing factors of postoperative recurrence in patients with pelvic organ prolapse.

Index	β	SE	Wald χ^2^	p	HR (95%CI)
Age	0.107	0.048	4.830	0.028	1.112 (1.012, 1.223)
Parturition times	0.698	0.315	4.906	0.027	2.009 (1.084, 3.724)
Mode of operation	0.424	0.731	0.336	0.562	1.528 (0.364, 6.407)
Continuous systolic blood pressure of type I muscle fibers	2.802	2.991	0.878	0.349	16.481 (0.047, 57.776)
Continuous systolic blood pressure of type II muscle fibers	-8.379	2.856	8.608	0.003	0.001 (0.001, 0.062)
Serum Elafin level	1.871	3.730	0.252	0.616	6.495 (0.004, 97.934)
Serum OPN level	-3.721	4.035	0.850	0.356	0.024 (0.001, 65.895)
Constant	41.108	27.821	2.183	0.140	–

CI: confidence interval; Elafin: elastase inhibitor; OPN: osteopontin; SE: standard error; HR: hazard ratio.

### Predictive model diagram of recurrence risk after pelvic organ prolapse

Based on the outcomes of logistic regression analysis, the software R4.1.3 was utilized to construct the line chart prediction model for estimating the chances of postoperative recurrence among patients with POP. Additionally, the ROC curve, calibration curve, and DCA curve were also generated. The findings indicate that the line chart prediction model exhibits a favorable level of discrimination, with an area under the ROC curve of 0.891 (95%CI 0.871–0.9921). Moreover, the calibration curve demonstrates a strong agreement between the ideal curve and the actual curve, underscoring the model’s accurate predictive ability. Furthermore, the DCA curve reveals that the model yields a higher net benefit value concerning the prediction of postoperative recurrence risk among POP patients.

## DISCUSSION

POP is a common condition among women, influenced by factors such as childbirth, age, obesity, and increased intra-abdominal pressure. It is particularly prevalent in elderly women due to a combination of these factors, with a prevalence rate of 9.6%. This condition significantly impacts women’s physical and mental well-being and quality of life^6,7^. The current standard treatment involves sacrum fixation, but postoperative recurrence rates are high, between 22 and 25%. Most research on recurrence focuses on postoperative factors, with limited discussion on preoperative factors. This study aims to explore preoperative conditions such as pelvic floor muscle strength, preoperative serological indicators, and uterine preservation to identify factors influencing postoperative recurrence and provide guidance for personalized surgical plans and perioperative treatments^8,9^.

Advanced age, obesity, and multiple deliveries are key factors contributing to POP. Older individuals with multiple deliveries have a higher likelihood of recurrence after surgery. Age-related declines in ovarian function reduce pelvic floor muscle elasticity, leading to recurrence. Childbirth stretches and damages the pelvic floor muscles, with greater parity causing more significant injury and a weaker postpartum recovery^10,11^. Pelvic floor muscles include type I and type II fibers. Type I fibers support the pelvic cavity, while type II fibers aid in pelvic and abdominal movement. The study collected data on the contraction strength and duration of these fibers, finding that reduced intensity and duration were linked to higher recurrence rates. Therefore, preoperative treatments to enhance muscle strength, careful surgical techniques, and postoperative exercises are recommended^12,13^. Further research is needed to determine critical preoperative muscle strength values indicating low recurrence risk.

POP’s etiology is linked to the extracellular matrix (ECM), with elastic fibers crucial for supporting pelvic floor tissue. Elafin, found in serum, inhibits inflammation and works with neutrophil elastase to maintain ECM stability. OPN is involved in fibrosis and ECM remodeling. The study measured serum Elafin and OPN levels, finding that lower levels were associated with higher recurrence risks. This information can guide clinical practice. Logistic regression analysis identified risk factors for recurrence, leading to the creation of predictive models using the R software. These models effectively predicted recurrence risk and had high clinical applicability, allowing early and accurate intervention to improve postoperative quality of life.

There is no standardized guideline on whether to preserve the uterus in POP patients. Some experts believe that uterine preservation can address psychological needs and maintain normal anatomical structures, promoting mental well-being^14-17^. However, some studies suggest that uterine preservation may increase recurrence risk. Our findings show a higher recurrence rate among patients who opted for uterine preservation compared to those who underwent hysterectomy. Nonetheless, uterine preservation is not an independent factor influencing recurrence, aligning with previous research. It is recommended to thoroughly evaluate the pros and cons of uterine preservation and hysterectomy, considering factors like age and pelvic muscle strength, to develop personalized surgical plans.

While our study provides valuable insights into the risk factors for postoperative recurrence in POP patients, several limitations must be acknowledged. First, the sample size of our study was relatively small, which may limit the generalizability of our findings. Future studies with larger cohorts are needed to confirm our results. Second, the retrospective nature of our study introduces potential biases, such as selection bias and recall bias. Prospective studies would provide more robust evidence. Third, the follow-up period was 3 years, which may not capture longer-term outcomes. Longer-term follow-up studies are required to assess long-term recurrence rates. Finally, while we have identified several risk factors, the complex interplay of these factors requires further investigation to fully understand their combined effects on recurrence. To build upon the findings of this study, future research could explore the role of adjuvant therapies, such as pelvic floor physiotherapy and lifestyle modifications, in reducing recurrence rates. Additionally, the clinical utility of the constructed risk prediction model should be further evaluated to inform surgical decision-making. By identifying patients at higher risk of recurrence, clinicians can tailor surgical strategies and perioperative care to minimize the risk of postoperative complications and improve patient outcomes.

In conclusion, factors such as age, delivery frequency, uterine preservation, and pelvic floor muscle strength during surgery impact POP recurrence. Advanced age, multiple deliveries, and sustained contraction and relaxation of type II muscle fibers are independent risk factors. Tailored surgical and perioperative treatment plans based on individual conditions are crucial to mitigate recurrence risk. Further confirmation of these findings with larger sample sizes is warranted.
